# Associations of maternal age with outcomes in very low birth weight singleton infants: a retrospective study

**DOI:** 10.3389/fped.2025.1444471

**Published:** 2025-02-26

**Authors:** Junfang Sun, Mengya Sun, Lulu Zhang, Chunchi Lai, Hong Jiang

**Affiliations:** Department of Neonatology, The Affiliated Hospital of Qingdao University, Qingdao, China

**Keywords:** maternal age, singleton, very low birth weight, infants, outcomes

## Abstract

**Background:**

With advances in perinatal medicine, there has been a rise in the preterm birth rate, especially the rate of very low birth weight (VLBW) and extremely low birth weight infants. Studies have shown that maternal age during pregnancy and at the time of delivery is associated with pregnancy complications and poor neonatal outcomes. Little is known about the effect of maternal age on the outcome of very low birth weight infants.

**Objectives:**

To investigate the effects of maternal age on the adverse outcomes of singleton very low birth weight neonates.

**Methods:**

We used data of VLBW infants from the neonatal database of our hospital. Maternal age was categorized as 20–24, 25–34 (reference group), 35–39 and ≥40 years. Statistical analyses included univariate and multivariate logistic regression analysis.

**Results:**

The study ultimately included 603 singleton, very low birth weight infants. After adjustment, neonatal outcomes in the group of older mothers were similar to those of the reference group for bronchopulmonary dysplasia, necrotizing enterocolitis, respiratory distress syndrome, severe asphyxia, retinopathy of prematurity and intraventricular hemorrhage grades 3–4. In the 20–24 year age group higher odds were present for sepsis [Odds ratio (OR) = 6.021; 95% confidence interval (CI), 1.741–20.818, *p* < 0.05] and for mortality (OR = 7.784; 95% CI, 2.198–27.568, *p* < 0.05). Higher odds for asphyxia (OR = 1.891; 95% CI, 1.238–2.890, *p* < 0.05) and death (OR = 2.101, 95% CI, 1.004–4.395, *p* < 0.05) were observed in infants of mothers in the 35–39 year age group. The incidence of sepsis was significantly higher in the age group of ≥40 years (OR = 2.873; 95% CI, 1.186–6.958, *p* < 0.05).

**Conclusions:**

In singleton very low birth weight neonates, neonatal outcomes were associated with maternal age, and adverse outcomes were more pronounced in infants of advanced maternal age (AMA) mothers.

## 1 Introduction

In the past few years, studies showed that maternal age during pregnancy and at the time of delivery was associated with pregnancy complications and poor neonatal outcomes. In particular, adverse neonatal outcomes were more prevalent at the extremes of maternal age, such as in teenage and advanced maternal age groups (1–3). For example, Cakmak et al. reported that mothers of extremely advanced maternal age and their newborns had similar perinatal and neonatal risks compared to younger mothers, except for lower 5th-minute Apgar scores (4). Xiong et al. showed a higher risk of gestational diabetes mellitus and cesarean delivery in mothers aged ≥35 years, compared to mothers aged <35 years. However, there was no significant difference in the risk of adverse neonatal prognosis among the mothers aged ≥35 years (5). Attali et al, found advanced maternal age to be an independent risk factor for intrapartum cesarean delivery and maternal age was not significantly associated with adverse neonatal outcomes (6). However, in addition to the above studies, most of the available studies on advanced maternal age included term or late preterm infants, and less was known about the very low birth weight infants (7, 8).

The aim of this study was to investigate the effect of maternal age on adverse perinatal and neonatal outcomes among very low birth weight infants born in the neonatal intensive care unit (NICU) of our hospital from January 2017 to December 2022. We hypothesized that at both ends of maternal age there were increased adverse neonatal outcomes in singleton very low birth weight infants.

## 2 Material and methods

### 2.1 Study design

This retrospective study was conducted on all live births between January 2017 and December 2022 with a birth weight ≤ 1,500 g. The study was approved by institutional research ethics committee of our hospital (QYFY WZLL 27383).

### 2.2 Patients

**Figure 1:**
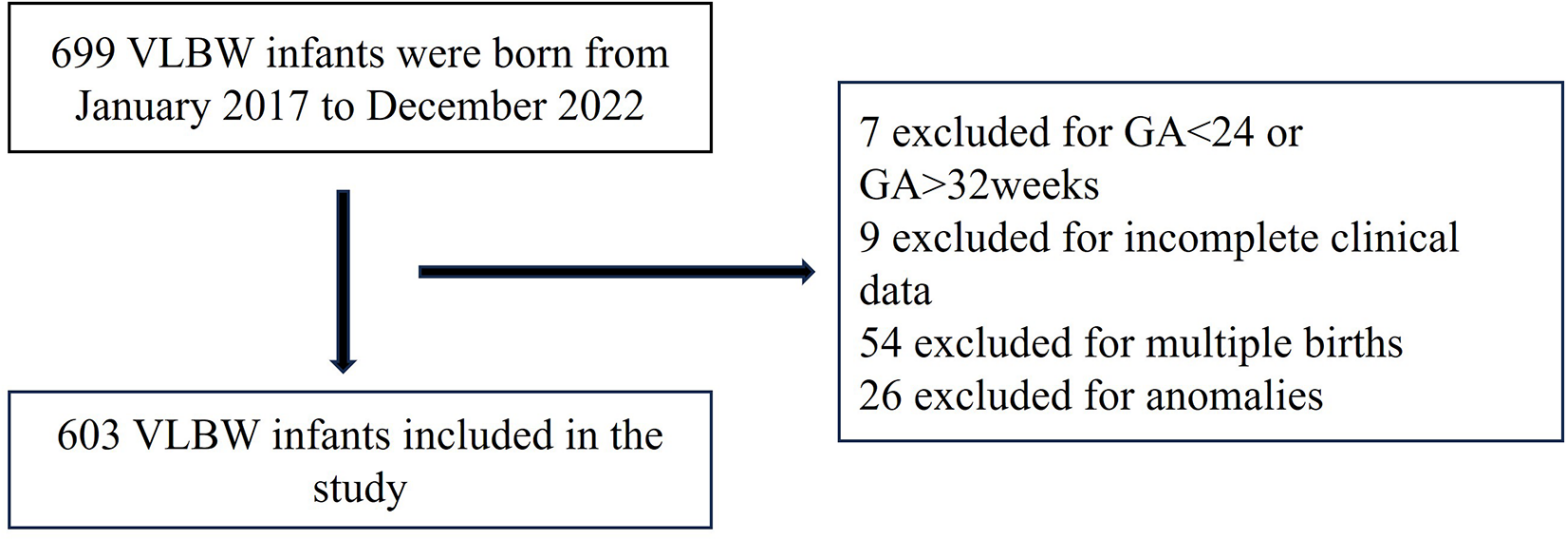
Flow chart of study population.

Between January 2017 and December 2022, 699 very low birth weight infants were enrolled. For the purpose of the study, we excluded infants <24 weeks' and ≥32 weeks' gestation (*n* = 7). As the study was limited to infants with birth weights ≤1,500 g, inclusion of infants ≥32 weeks' gestation would have resulted in an over-representation of small for gestational age (SGA) infants. We also excluded infants with incomplete clinical information and those with severe congenital malformations (*n* = 35). The latter group was excluded in order to focus only on prematurity and low birth weight without increasing the confounding effect of severe congenital malformations. In addition, we excluded all multiple births (*n* = 54), an important confounder in neonatal outcome studies. The final study population consisted of 603 singleton, very preterm, very low birth weight infants (Flow chart, Figure 1).

### 2.3 Definitions

The neonatologist defined gestational age (GA) in completed week as the best estimate based on the reported last menstrual period, obstetrical history and examination, and the date determined from an early (first trimester) prenatal ultrasound. Birth weight (BW) percentile was determined using gender-specific charts of Kramer et al. (9). Birth weights less than the 10th percentile were defined as SGA. Prenatal steroid treatment included partial and full courses. Neonatal adverse outcomes included: bronchopulmonary dysplasia (BPD), necrotizing enterocolitis (NEC), sepsis, respiratory distress syndrome (RDS), asphyxia, intraventricular hemorrhage (IVH) grades 3–4, retinopathy of prematurity (ROP) and mortality. RDS was diagnosed by typical chest radiograph findings and required supplemental oxygen or mechanical ventilation (10). Asphyxia was diagnosed according to the criteria established by the Chinese Neonatal Asphyxia Group (11). BPD was diagnosed according to the diagnostic criteria (12), including clinical and radiological features and the need for oxygen therapy even after correction of gestational age of 36 weeks. IVH was diagnosed and graded according to Papile's study (13). NEC was determined by the clinical and radiological criteria of Bell et al. (14). Sepsis was diagnosed when the pathogen was isolated from blood or cerebrospinal fluid and antibiotic therapy was given for ≥5 days. Perinatal characteristics, including gestational diabetes, gestational hypertension, premature rupture of membranes (PROM), were defined in accordance with the American College of Obstetricians and Gynecologists (ACOG) guidelines (15–17). The classic definition of placental abruption is complete or partial separation of the normally implanted placenta prior to delivery (18).

### 2.4 Statistical analysis

The cohort was categorized into maternal age groups according to completed years: 20–24 years, 25–34 years, 35–39 years and ≥40 years. Statistical analysis was performed using SPSS statistical software version 26.0. To compare the distribution of maternal perinatal factors and neonatal morbidity in each group, categorical variables were tested using chi-square test or Fisher's exact test. Continuous variables that did not conform to normal distribution were tested using the Kruskal–Wallis H test. Multivariate logistic regression analyses were performed to assess the independent effect of maternal age group on mortality and neonatal morbidity. The results of the logistic models were analyzed with appropriate 95% confidence intervals for the adjusted odds ratios (OR). A *p*-value less than 0.05 was statistically significant.

## 3 Results

The main demographic, gestational, and perinatal characteristics by maternal age group were presented in Table 1. An increase in maternal age was associated with an increase in the proportion of cesarean sections (*P* < 0.05). Table 2 showed the mortality and neonatal morbidity rates for singleton very low birth weight infants by maternal age group. Increasing maternal age was also associated with higher rates of mortality (*P* < 0.001), sepsis (*P* < 0.05), and asphyxia (*P* < 0.05), whereas rates of ROP, IVH, BPD, and NEC were similar across groups.

**Table 1:** Baseline clinical characteristics of singleton VLBW infants by maternal age groups (*N* = 603).

Clinical characteristics	Maternal age group (years)				*P* value
	20–24 *N* = 15 *n* (%)	25–34 *N* = 315 *n* (%)	35–39 *N* = 213 *n* (%)	≥40 *N* = 60 *n* (%)	
Birth weight, g	1,140 [940, 1,200]	995 [920, 1,220]	998 [900, 1,250]	990 [903, 1,200]	0.843
Gestational age, weeks	28.6 [28.0, 29.6]	28.6 [27.7, 29.4]	28.9 [28.0, 29.6]	28.6 [28.0, 29.7]	0.092
SGA	2 (13.3)	41 (13.0)	31 (14.6)	11 (18.3)	0.728
Male	8 (53.3)	175 (55.6)	116 (54.5)	28 (46.7)	0.657
Cesarean delivery	7 (46.7)	130 (41.3)	132 (62.0)	42 (70.0)	<**0**.**001**
PROM	0 (0)	0 (0)	1 (0.5)	1 (1.7)	0.129
Maternal hypertensive disorders	1 (6.7)	14 (4.4)	15 (7.1)	4 (6.7)	0.481
Preeclampsia	1 (6.7)	14 (4.4)	15 (7.1)	4 (6.7)	0.477
Maternal diabetes	0 (0)	42 (13.3)	37 (17.4)	9 (15)	0.246
Meconium-stained amniotic fluid	2 (13.3)	42 (13.3)	30 (14.1)	11 (18.3)	0.797
Placental abruption	3 (20.0)	19 (6.0)	22 (10.3)	4 (6.7)	0.084
Antenatal steroids	7 (46.7)	112(35.6)	73(34.3)	26(43.3)	0.490

Statistically significant values are in bold.

**Table 2:** Neonatal morbidities and mortality among singleton VLBW infants by maternal age groups (*N* = 603).

Clinical characteristics	Maternal age group (years)				*P* value
	20–24 *N* = 15 *n* (%)	25–34 *N* = 315 *n* (%)	35–39 *N* = 213 *n* (%)	≥40 *N* = 60 *n* (%)	
BPD	3 (20.0)	69 (21.9)	54 (25.4)	15 (25.0)	0.807
NEC	1 (6.7)	9 (2.9)	7 (3.3)	2 (3.3)	0.626
Sepsis	4 (26.7)	18 (5.7)	14 (6.6)	9 (15.0)	**0**.**005**
RDS	5 (33.3)	127 (40.3)	108 (50.7)	28 (46.7)	0.093
Asphyxia	7 (46.7)	130 (41.3)	132 (62.0)	42 (70.0)	**0**.**011**
Severe asphyxia	0 (0)	4 (1.3)	4 (1.9)	1 (1.7)	0.761
IVH Grade 3–4	1 (6.7)	15 (4.8)	10 (4.7)	2 (3.3)	0.876
ROP	1 (6.7)	22 (7.0)	17 (8.0)	3 (5.0)	0.895
Mortality	4 (26.6)	14(4.4)	18(8.5)	1(1.7)	**0**.**003**

Statistically significant values are in bold.

The results of the multivariate logistic regression analyses were presented in Table 3. Mothers in the 25–34 years age group were considered the reference group for these analyses. In the adjusted model, the incidence of sepsis (OR = 6.021; 95% CI, 1.741–20.818, *p* < 0.05) and mortality (OR = 7.784; 95% CI, 2.198–27.568, *p* < 0.05) were higher in mothers aged 20–24 years. Mothers aged 35–39 years had a higher incidence of infant asphyxia (OR = 1.891; 95% CI, 1.238–2.890, *p* < 0.05) and death (OR = 2.101, 95% CI, 1.004–4.395, *p* < 0.05). Infants of mothers aged ≥ 40 years had a significantly higher incidence of sepsis (OR = 2.873; 95% CI, 1.186–6.958, *p* < 0.05).

**Table 3:** Adjusted OR and 95% CI for mortality and morbidities among singleton VLBW infants by maternal age groups.

Outcome	Maternal age group (years)			
	20–24 OR (95% CI)^a^	25–34^b^ Reference	35–39 OR (95% CI)^a^	≥40 OR (95% CI)^a^
Sepsis	**6.021** **(1.741–20.818)**	1.0	1.131 (0.542–2.363)	**2.873** (**1.186–6.958)**
Asphyxia	0.708 (0.155–3.229)	1.0	**1.891** (**1.238–2.890)**	0.788 (0.361–1.720)
Mortality	**7.784** **(2.198–27.568)**	1.0	**2.101** (**1.004–4.395)**	0.379 (0.048–3.010)

Statistically significant values are in bold.

^a^ Adjusted for birth weight, gestational age, SGA, sex, mode of delivery, PROM, maternal hypertensive disorders, and maternal diabetes.

^b^ Reference group.

## 4 Discussion

In this retrospective study based on very-low-birth-weight infants, our main finding was that, after adjustment, infants of older mothers had significantly more adverse outcomes. In fact, there were significant differences in prognosis for a specific maternal age group compared to the reference age group (25–34 years): sepsis (higher odds in the lowest maternal age group and in the ≥40 years group) and mortality (higher odds in the 20–24 and 35–39 years groups). Asphyxia was more frequent in the group 35–39 years.

Cesarean section rates were significantly different among age groups in univariate analysis. Older women were more likely to deliver by cesarean section. The reasons for this finding may be multiple. On the one hand, in China, the two-child policy was liberalized from January 1, 2016, which means that a couple can have two children. This has led to a number of repeat cesarean deliveries among older women. On the other hand, the demand for cesarean delivery by women with “precious” pregnancies was also a reason, such as the advanced age of first-time mothers after multiple attempts of infertility treatment. In addition, career advancement, which sometimes requires longer years of higher education, and other personal reasons may lead to delayed marriage and delayed age at conception. Some studies have shown that the high rate of cesarean section in highly educated women is due to the increase in pregnancy and labor complications brought about by the age factor as a result of educational experience (19). In the United States, statistics showed that in 2005 and 2006, the birth rate among women aged 40–44 continued to rise, reaching 9.4 per 1,000 live births (20–22).

Our study focused on neonatal complications in very low birth weight infants of mothers of different ages. Most articles examining the effects of advanced maternal age or teenage pregnancy on neonatal outcomes study the general neonatal population and do not specifically address very low birth weight infants.

In a 2019 study, Tseng et al. found that in a cohort of 536 very low birth weight infants born to 483 mothers, there was no significant difference in short-term neonatal outcomes between the group born to mothers of AMA and the control group (7). In a study published by DiLabio in 2020 (8), data from the Canadian Neonatal Network noted the effect of maternal age on long-term outcomes of preterm infants with gestational age less than 29 weeks. In this study, in univariate analyses, most short-term outcomes were not affected by advanced maternal age. In another 2020 study, data showed that adverse fetal outcomes, including stillbirth and preterm birth, were significantly associated with advanced maternal age, but low birth weight was not significantly associated with advanced maternal age (23). However, this study did not mention the effect of advanced maternal age on early postnatal morbidity in newborns. The study by Sunil et al. focused on evaluating the effect of increasing maternal age on maternal and neonatal Outcomes in pregnancies at advanced maternal age. The rates of preterm birth and NICU admission increased significantly with increasing maternal age at advanced maternal age (24). In an additional study published in 2022, Shen et al. found that higher maternal age seemed associated to the risk of switching to emergency cesarean section and having abnormal fetal heart rate. A major limitation of the study was the lack of control for potential clinical covariates that may bias the effects of maternal age (25). In Qi's study (26), data from the Chinese Neonatal Network (CHNN) showed that in very preterm infants, increased maternal age was associated with a higher incidence of small for gestational age but not neonatal mortality. Young maternal age may increase the risk of severe intraventricular hemorrhage in very preterm infants. In a 2023 study, Kasirer et al. found that neonatal outcomes among very low birth weight infants were associated with maternal age, and adverse outcomes were more pronounced in infants of younger mothers (27).

The main strength of this study was that there are currently no related studies reported in China. In addition, the study was based on a neonatal database from a university-affiliated hospital in China. However, several limitations should be considered when evaluating the results of this study. First, our population consisted mainly of people from the peninsular area of Shandong province, with few inland areas. Therefore, our results may not necessarily apply to all groups in the province. In addition, because we excluded multiple births, an important confounder in neonatal outcome studies, the results of this study may apply only to singleton very low birth weight infants. Another limitation was that we were unable to assess the effect of socioeconomic status on outcomes, as this information was not included in the database.

## 5 Conclusion

We conclude that neonatal outcomes are not identical for singleton very low birth weight infants, which are influenced by maternal age. Neonatal outcomes tend to be poorer in deliveries to mothers aged ≥35 years. The results of this study can help provide information about neonatal outcomes for mothers of newborns with VLBW in different age groups. We hope that in the coming years there will also be descriptions of long-term outcomes for VLBW infants and better perinatal counseling services for mothers.

## Data Availability

The original contributions presented in the study are included in the article/Supplementary Material, further inquiries can be directed to the corresponding author.
